# Recent Molecular Tools for the Genetic Manipulation of Highly Industrially Important Mucoromycota Fungi

**DOI:** 10.3390/jof7121061

**Published:** 2021-12-10

**Authors:** Hassan Mohamed, Tahira Naz, Junhuan Yang, Aabid Manzoor Shah, Yusuf Nazir, Yuanda Song

**Affiliations:** 1 Colin Ratledge Center for Microbial Lipids, School of Agriculture Engineering and Food Science, Shandong University of Technology, Zibo 255000, China; hassanmohamed85@azhar.edu.eg (H.M.); nazkhan658@gmail.com (T.N.); judywoniu@163.com (J.Y.); aabidshah@sdut.edu.cn (A.M.S.); yusufnazir91@yahoo.com (Y.N.); 2 Department of Botany and Microbiology, Faculty of Science, Al-Azhar University, Assiut 71524, Egypt; 3 Department of Food Sciences, Faculty of Science and Technology, University Kebangsaan Malaysia, UKM Bangi 43600, Malaysia

**Keywords:** mucorales fungi, genetic modifications, model organisms

## Abstract

Mucorales is the largest and most well-studied order of the phylum Mucormycota and is known for its rapid growth rate and various industrial applications. The Mucorales fungi are a fascinating group of filamentous organisms with many uses in research and the industrial and medical fields. They are widely used biotechnological producers of various secondary metabolites and other value-added products. Certain members of Mucorales are extensively used as model organisms for genetic and molecular investigation and have extended our understanding of the metabolisms of other members of this order as well. Compared with other fungal species, our understanding of Mucoralean fungi is still in its infancy, which could be linked to their lack of effective genetic tools. However, recent advancements in molecular tools and approaches, such as the construction of recyclable markers, silencing vectors, and the CRISPR-Cas9-based gene-editing system, have helped us to modify the genomes of these model organisms. Multiple genetic modifications have been shown to generate valuable products on a large scale and helped us to understand the morphogenesis, basic biology, pathogenesis, and host–pathogen interactions of Mucoralean fungi. In this review, we discuss various conventional and modern genetic tools and approaches used for efficient gene modification in industrially important members of Mucorales.

## 1. Introduction

Mucoromycota comprise a diverse group of molds, including the common bread molds, such as *Mucor* and *Rhizopus*. The phylum Mucoromycota consists of three subphyla:

(1) the Glomeromycotina, which form arbuscular mycorrhizae,
(2) the Mortierellomycotina, which are typically root endophytes, and,
(3) the Mucoromycotina, which include the orders Mucorales, Umbelopsidales, and Endogonales [1].

Mucorales is a fascinating group of filamentous fungi and the largest and most well-studied order of Mucoromycota, which is currently poorly recognized due to polyphyly. According to earlier morphological classifications, fungi reproducing via zygospores were classified in the phylum Zygomycota [2]. However, the phylum Zygomycota was abandoned because it was not supported in several molecular phylogenies that included a higher number of loci and taxa [3], a fact that resulted in many incorrect citations [1]. On the basis of recent and confirmed results, the phylum Mucoromycota was defined [4].

Members of the order Mucorales are occasionally described as pin molds. Mucoralean fungi are known for their rapid growth and large hyphae (long, filamentous structures) that lack septa. Most of the hyphae develop within the substrate. Sporangiophores are sac-like sporangia packed with asexual sporangiospores that are supported by upright hyphae. Mucorales are haploid microorganisms that normally reproduce asexually via sporangiospores; however, they are heterothallic, and can also reproduce sexually via zygospores, where meiosis takes place [5]. For reproduction via zygospores one (+)-strain and one (−)-strain are needed. Numerous fungi within Mucorales have been associated with human pathogenesis, while others are related to food and industry. A few members of the order Mucorales, such as *Mucor*, are dimorphic fungi, capable of isotropic and polarized forms; depending on the environmental conditions, they can grow as yeast or hyphae respectively [6]. Their predominant growth form is filamentation, but certain conditions promote yeast growth [7].

The calcineurin gene was found to be involved in the regulation of this dimorphism process [7,8]. This process was also found to contribute to virulence in species of the order Mucorales [7]. Fungi in the Mucorales order have shown many uses in research and in industrial and medical fields. For instance, *Mucor* species are used in biotechnology as bio-transformants to produce useful enzymes and diverse metabolites [9]. Additionally, some Mucorales species are biotechnological producers of extracellular hydrolytic enzymes (lipases and proteases) [10], organic acids such as fumaric, malic and lactic acid [11,12], alcohol and carotenoids [13,14], and steroids and terpenoids [5], while others are employed in oriental food fermentation processes as starter cultures [15]. Moreover, several of these species acts as plant parasites or spoilage organisms, and can cause life-threatening infections such as mucormycosis [1], which is known as the second-most common fungal deep mycosis after Aspergillosis [16]. Furthermore, certain members of Mucorales, such as *Rhizopus arrhizus*, *Phycomyces blakesleeanus* and *M. circinelloides*, are extensively used as model organisms for genetic and molecular investigation, including in light sensing [17], signal processing and molecular regulation [18], morphogenesis [19], sexual reproduction and differentiation [20], pathogenicity [21,22], and carotenogenesis [23].

Kosa et al. recently studied more than 100 strains of Mucoromycota and found that most of them showed a filamentous growth mode [24]. However, certain members of the order Mucorales could produce yeast, spores, and mycelia in response to environmental conditions [25]. It has been observed that the absence of oxygen and availability of hexose sugars can stimulate yeast growth while aerobic conditions can induce hyphal growth [26]. Genetic manipulation tools, such as gene deletion constructs, have identified genes that influence morphogenesis, and this information has led to the identification of interesting candidates for therapeutic development. For instance, the role of the calcineurin pathway in regulating the yeast–mycelium transition and virulence was determined in *M. circinelloides* by homologous recombination of upstream and downstream sequences flanking an auxotrophic marker such as *pyrG* [8]. Disruption of the CnbR gene (or the addition of calcineurin inhibitors) locked the fungus in yeast form, making it significantly less virulent in mice. In addition to calcineurin, the cyclic AMP (cAMP) pathway and its target protein kinase A (PKA) reportedly participate in controlling morphogenesis. Interestingly, calcineurin inhibits PKA, implying a connection between these two regulatory mechanisms [7]. Related gene deletions, including the deletion of heterotrimeric G proteins and ADP-ribosylation factors (Arfs), revealed other proteins involved in the control of *M. circinelloides* dimorphism [27].

Despite all these important contributions, *Mucor* spp. are less investigated than basidiomycetous or ascomycetous species, and their taxonomy, metabolism and ecology are less documented than their counterparts among the higher fungi [5]. However, scientists are beginning to make progress in overcoming the challenges of understanding Mucorales genetics. Although we are still far from fully comprehending the Mucorales, various methods for developing new tools to investigate the order have been documented in the literature [28]. Researchers have mainly focused on *M. circinelloides*, which has expanded the biological understanding of the genus and fungal biology in general, with particular advancements in the understanding of fungal dimorphism and light-mediated gene regulation [5].

## 2. Attributes of Model Organisms for Genetic Manipulation

The development of suitable and stable genetically modified organisms to serve as hosts and express desired target genes is necessary for successful molecular genetic analysis and protein synthesis. A model organism is studied to improve our knowledge of biological processes. Model organisms are appealing because they are industrially important, contain a broad genetic toolbox that permits rapid and simple modifications, and have been the subject of decades of research. However, transforming synthetic pathways into model organisms is often most successful with short heterologous pathways, whereas long synthetic and heterologous pathways are generally less resilient and can create a substantial metabolic burden.

The most common characteristics of model organisms include a short lifespan, an available genomic sequence, being well understood, and the abilities to grow quickly, to be easily controlled, and to produce a high number of offspring. They should be easy to store in a laboratory and non-pathogenic [29]. They must also resemble other organisms or systems. There are three different types of model organisms: genetic, genomic, and experimental.

- Genetic model organisms are species that can be genetically modified, and large-scale genetic crosses are possible with them;
- Genomic model organisms have a particular genomic size or composition that can be used, such as pufferfish;
- Experimental model organisms may not be amenable to genetic modification; however, other advantages are specific to the experiment and the traits being sought.

The completion of the human genome project required the use of model organisms. These were used to create novel DNA sequencing technology, data analysis, and project management techniques [30]. Model organisms are also crucial in the development of metabolomics techniques [31]. A trade-off in the selection of a model organism is whether it can display the desired phenotype. The selection of organisms should also reflect the level of the research question. When researchers choose a model organism, they consider many characteristics, including size, generation time, accessibility, mechanism conservation, manipulation, genetics, and possible economic gain.

Certain members of Mucorales, such as *R. arrhizus*, *P. blakesleeanus* and *M. circinelloides*, are extensively used as model organisms for genetic and molecular investigation. The order Mucorales contains a few industrially important model organisms that are well-studied and amenable to genetic modification. Some of these are also used to investigate and understand pathogenesis and host–pathogen interactions in mucormycosis. Nevertheless, genetic understanding of Mucorales remains challenging due to the lack of dominant selection markers, limited transformation efficiency, and low frequency of chromosomal integration [32]. Therefore, our understanding of the genetics of most Mucorales species remains limited. Whole-genome duplication occurred early in the Mucoromycotina lineage and may have produced additional proteins that expanded the signaling and sensory pathways [33]. Therefore, in this review, we consider a few industrially important Mucorales model organisms due to the availability of genetic tools for them. We also highlight a few important genetic modification tools and approaches that are used to manipulate the Mucorales genome for useful metabolite production and gene regulatory studies. The following are the most well known Mucoromycota model organisms employed in various industries for bioactive compound production due to their ease of genetic manipulation. 

### 2.1. Mucor circinelloides

Although the genetic study of the *Mucor* genus is at a very early stage compared with that of *Neurospora* or *Aspergillus*, this genus has unique characteristics that make it a source of desirable model organisms for genetic study and basic and applied research. A 36.6 Mb genomic sequence of *M. circinelloides* f. *lusitanicus* was the first publicly available *Mucor* genome (http://genome.jgi.doe.gov/Mucci2/Mucci2.home.html (accessed on 20 June 2016.) and the ongoing annotation of this fungus will help in deciphering its metabolism. It is also an attractive target for biotechnological development, due to its dimorphic nature [34] and the availability of an efficient transformation system, allowing improved carotene content production [35].

Van Heeswijck and Roncero (1984) were the first researchers to transform the protoplast of *M. circinelloides* with plasmids using CaC_l2_ and a polyethylene glycol-based method [36,37]. Since then, the process of transformation has been improved regularly, including the introduction of strategies such as transformation mediated by *Agrobacterium tumefaciens* (ATMT) [38] and biolistic [39] mediated transformation, but protoplast mediation remains the gold standard protocol for *Mucor* transformation [37]. Previously, self-replication plasmids were used for transformation in *M. circinelloides* [40]. Similarly, genomic integration could also be carried out either with ATMT [38] or restricted enzyme-mediated transformation (REMI) [41]. The availability of genetic tools and the ability to knock out desired genes in *M. circinelloides* has permitted important breakthroughs in fungal genetics, particularly in gene regulation [28].

Though *Neurospora crassa* can be a model for understanding blue light regulation, several reports on *M. circinelloides* have also proven highly informative to mycologists. Because it is dimorphic, this species is considered an excellent candidate to illuminate the transition from yeast to hyphal growth via mutants [7]. More specific progress has been made in understanding how the RNA-mediated gene silencing pathway or RNA interference (RNAi), a conserved defense mechanism against invasive nucleic acids, may regulate genes. The RNAi machinery of *M. circinelloides* not only functions as a defense mechanism but also plays an important role in the regulation of many endogenous genes [42].

*M. circinelloides* has also been exploited for enzymes, carotenoids [14], and other metabolites, and to understand the genetic and molecular foundations of Mucorales fungi’s pathogenicity [7,43]. *M. circinelloides* is considered a true “microbial factory” due to its intrinsic potential to produce enzymes, organic acids, essential amino acids, food pigments, ethanol, chitosan, polyphenols, and microbial lipids [44,45]. For instance, *M. circinelloides* can produce the nutritionally important polyunsaturated fatty acid (PUFA) gamma-linolenic acid (GLA, C18:3), which supports animal and human health. *M. circinelloides* is a pioneer microorganism used for commercial GLA production for human consumption, and has attracted much research attention as a result. Kabir et al. carried out the genetic modification of *M. circinelloides* for dihomo-gamma-linolenic acid (DGLA) production. They overexpressed the delta-6 elongase gene from *M. alpina* and found 5.72% DGLA production in the modified strain [46].

*M. Circinelloides,* being model organisms for carotenogenic studies, have also been employed for heterologous production. Previously, Papp et al [35] successfully inserted the *crtW* gene from a marine bacterium into *M. circinelloides* and produced canthaxanthin (6–13 μg/g) and astaxanthin (3 µg/g) [35]. To enhance astaxanthin production in *Mucor* spp., some researchers expressed the *Paracoccus crtZ* gene in canthaxanthin-producing *M. circinelloides* mutants. They obtained a maximum of 443 ± 71 μg/g of canthaxanthin and 14–35 μg/g of astaxanthin when the mutant was grown on an optimized medium [47]. Csernetics et al. also genetically manipulated *M. circinelloides* by introducing *crtR* and *crtS* from *X. dendrorhous*, a yeast, resulting in 190 μg/g of canthaxanthin [48].

### 2.2. Rhizopus arrhizus

The fungus *R. arrhizus* (and its close relative *R. delemar*) is a common filamentous fungus found on decaying organic matter. It belongs to the Mucorales order of the phylum Mucoromycota. *Rhizopus stolonifer*, formerly known as *Rhizopus nigricans*, was the first species to be described in the genus *Rhizopus*, and it is recognized for producing fermentation products such as ethanol, L(+)-lactic acid, fumaric acid, and L(+)-malic acid, to a lesser extent [49]. In Asia, *R. arrhizus* strains are commonly employed for food fermentation (as *R. arrhizus* is generally recognized as safe) to produce alcoholic drinks, ragi, or tempeh, and the strains are usually considered harmless. *R. arrhizus* is also an opportunistic human pathogen with a high incidence in mucormycosis infections [50]. *R.* *arrhizus* strains can be classified into two types based on the primary organic acid produced when grown on D-glucose [51]. The first group mainly produces lactic acid, and the second mainly fumaric and L(+)-malic acids. High yields of these chemicals are produced on a wide range of carbon sources, with L(+)-lactic acid and ethanol yields exceeding 85% of the theoretical yield and fumaric acid yields above 65% of the theoretical yield. The genome of *R. arrhizus* strain 99–880 (a type II strain) was published in 2004. This was a significant contribution and provided fresh insights into molecular approaches, as it was the first of the polyphyletic zygomycetes to be sequenced. Because of the whole-genome duplication, gene deletion and silencing techniques to genetically modify pathways are challenging to implement [49].

Recently, our understanding of heterologous gene expression in *R.* *arrhizus* has improved dramatically and three transformation systems have been identified: *Agrobacterium tumefaciens*-mediated transformation (ATMT) [52], protoplast generation and transformation with exogenous DNA via the CaCl_2_/PEG method [52], and a particle bombardment DNA delivery system [53]. Various metabolic engineering tools have recently become available, including random mutagenesis, RNAi, and gene knockout strategies. Furthermore, it is now possible to incorporate heterologous genes, leading to the development of new products and, eventually, complete pathways. Multiple studies have reported that various auxotrophic selection markers should be established to allow multiple-gene introduction. This should be combined with more research to discover the fate of the injected DNA, as it seldom integrates into the genome. In short, *R. arrhizus* is a versatile organism that has already been employed for a variety of purposes, and is expected to be used for more in the future [49].

### 2.3. Mortierella alpina

The order Mortierellales, which is part of the Mortierellomycotina subphylum (phylum: Mucoromycota), contains the Mortierellaceae family, which consists of more than 80 species in five genera. *Mortierella* species are soil-inhabiting fungi [4]. Mortierellales members are ecologically and physiologically varied, allowing them to be found all over the planet. *M. alpina* is an oleaginous filamentous fungus with a high capacity for fatty acid biosynthesis that may produce a wide range of biologically active PUFAs, such as arachidonic acid (ARA) and eicosapentaenoic acid (EPA), at an industrial scale [54]. *Mortierella* fungi (Mortierellomycotina, Mortierellales) are normally coenocytic; however, they have a stronger inclination toward septum formation than *Mucor* fungus. *Mortierella* species are typically non-pathogenic towards humans, plants, and animals. *M. alpina* was identified as a good candidate for the commercial production of high-value PUFAs in 1987, due to its triacylglycerol profiles abundant in high-value PUFAs [55]. Because of its high ARA content (>50% of its total fatty acid content), the lipid fraction generated by *M. alpina* is often utilized as a dietary supplement. The US Food and Drug Administration (FDA) has certified ARA-rich oils extracted from *M. alpina* as generally recognized as safe (GRAS). *M. alpina* can produce both n-3 and n-6 PUFAs and the yields can be controlled via fermentation and genetic manipulation.

This biotechnological importance has sparked interest in studying *M. alpina* lipid metabolism in order to leverage the species to generate certain fatty acids [56]. Since an efficient genetic toolbox was introduced, biotechnology-enabled genetic manipulation of *M. alpina* has increased its lipid accumulation and PUFA synthesis capacities [57,58]. Recent developments in omics technology and genetic engineering have exposed various critical parameters that regulate lipid yield and PUFA production in *M. alpina*, allowing genetic modification and the development of fermentation techniques [59,60]. A transcriptome analysis revealed that the expression pattern of lipogenesis-related genes in *M. alpina* was quite comparable to those of other oleaginous fungi [61,62]. Combining mutation or genetic modification with fermentation optimization is still a popular strategy for increasing *M. alpina*’s lipid yield [63,64]. Breeding and genetic engineering require research into the biosynthesis of ARA and related critical enzymes.

*M. alpina* has been effectively genetically transformed in the past few years. In 2000, Mackenzie published the first report on a *M. alpina* transformation system. Using the homologous histone (hisH4.1) promoter, the hygromycin resistance gene was transformed within the protoplast of *M. alpina* CBS 224.37 to produce a resistant strain using the traditional polythene glycol approach [65]. In addition, Ando et al. established the microprojectile bombardment and ATMT methods, which resulted in a transformation frequency of more than 400 transformants per 108 spores and mitotic stability in 60 to 80% of the transformants [57,66]. DNA from an exogenous source is integrated into the chromosome via homologous recombination (HR) and non-homologous end-joining (NHEJ) DNA double-strand break repair processes. The HR technique is frequently used for gene targeting, precisely introducing foreign genes to destroy target genes. Eliminating the critical proteins involved in NHEJ has been reported to improve efficiency [67].

Several genes involved in fatty acid synthesis and reducing power were found to be overexpressed in *M. alpina.* In one overexpression study, Δ12 desaturase was overexpressed in *M. alpina* JT-180 and ARA production and proportion were enhanced by 66.7% and 18% of total fatty acids, respectively, compared with wild-type strain 1S-4 [68].

Based on the understanding that increasing the supply of the reducing power NADPH could improve lipid and ARA production in *M. alpina*, Hao et al. overexpressed the malic enzymes malE1 and malE2, glucose-6-phosphate dehydrogenase (G6PD2), and 6-phosphate gluconate dehydrogenase in *M. alpina*. Overexpression of G6PD2 resulted in a 1.7-fold rise in total fatty acid production, while malE2 overexpression resulted in a 1.5-fold increase in ARA content [60]. In another study, the gamma-linolenic acid elongase gene, which encodes the fatty acid elongase EL2 that catalyzes the conversion of gamma-linolenic acid into dihomo-gamma-linolenic acid, was successfully introduced into a target strain of the fungus. This resulted in a twofold ARA production increase compared with the parent strain [69]. Hao et al. genetically manipulated the fungus to improve its utilization of glycerol. They overexpressed glycerol kinase (GK) and glycerol-3-phosphate dehydrogenase (G3PD) and found that G3PD did not significantly affect lipid accumulation, but GK improved the total fatty acid content by up to 35%. The co-overexpression of GK and malic enzyme (ME1) was found to increase fatty acid accumulation by 81% in *M. alpina* when it was cultivated with pure glycerol. They observed that ARA yield increased by 60% when the repeat batch approach was used to overcome the inhibitory effect of excessive raw glycerol content [70].

The aerobic fatty acid desaturase/elongase pathway catalyzes PUFA synthesis in *M. alpina* and requires exceptionally high oxygen consumption. Sufficient dissolved oxygen (DO) has been shown to play an essential role in enhancing the degree of fatty acid unsaturation [59]. One recent study on *M. alpina* ATCC 32222 promoted the heterologous overexpression of the *VHb* (*Vitreoscilla hamoglobin*) gene to boost the utilization of oxygen, and found that the modified strain produced four to eight times more total lipids and ARA than the original strain [71]. To investigate the function of beta-isopropylmalate dehydrogenase (IPMDH) in lipogenesis, Tangs et al. executed the homologous overexpression of *MaLeuB*, the gene that encodes IPMDH. They found that the total fatty acid content of the recombinant strain was 20.2% higher than that of the control strain, representing a fourfold increase in the *MaLeuB* transcriptional level [54].

### 2.4. Cunninghamella spp.

*Cunninghamella* are oleaginous fungi in the order Mucorales and the *Cunninghamellaceae* family. They are filamentous fungi that can be found in soil and plant matter, especially in Mediterranean and subtropical climates [72,73]. They have also been found in animal tissue, cheese, and Brazil nuts [74]. Because species of this genus metabolize a wide range of medications in ways that are similar to mammalian enzyme systems, members of this genus are frequently employed to investigate drug metabolism in an in vitro model [75]. *Cunninghamella* species use both phase I (oxidative) and phase II (conjugative) biotransformation processes to digest a wide array of xenobiotics [76]. This species is frequently utilized as a microbial model for the metabolism of aromatic xenobiotics in mammals [77]. More than 20 species are found in the genus *Cunninghamella*, among which *C. elegans*, *C. bertholletiae*, and *C. echinulata* are the most common species. However, other species were reported to have been studied in a majority of scientific surveys, including *C. blakesleeana*, *C. echinulata* var. *echinulata*, *C. clavata*, *C. echinulata* var. *nodosa* var. nov., *C. intermedia, C. homothallica*, *C. septata* sp. nov., and *C. vesiculosa*.

*C. echinulata* is frequently found as an air contaminant [78], and recently attracted the interest of the biotechnology industry because of its potential to produce γ-linolenic acid and its ability to bioconcentrate metals [79]. *C. echinulata* prefers a nitrogen-depleted medium with a C/N molar ratio of 169 to produce the greatest amount of GLA [80]. The literature reported that given a high C/N ratio of more than 100, *C. echinulata* could produce over 35% *w*/*w* cellular storage lipids and over 11% *w*/*w* GLA. GLA content in *C. echinulata* steadily increased after the accumulation of cellular lipids, with the greatest GLA yield up to 720 mg/L; 80 mg/g of dry biomass was reported with a C/N ratio of 163 [80]. Various studies have also found that ideal or optimized conditions are crucial for lipid quality and output and that GLA yield varied by dietary source. For instance, when *C. echinulata* ATHUM4411 was grown with tomato waste hydrolysate, it yielded 8.7 g/L of lipids and 1018 mg/L of GLA; potato starch yielded 3.8 g/L of lipids and 540 mg/L of GLA [81,82]. Using starch as a substrate, *C. echinulate* CCRC 31840 produced 964 mg/L of GLA, which could be enhanced to 1349 mg/L by optimizing the inoculum [83]. This fungus is now widely grown for its ability to generate GLA, with a preference for R- p-hydroxymexiletine (R-PHM) and S-hydroxymethyl mexiletine (S-HMM) syntheses [84]. The growth conditions for this strain to produce GLA have been thoroughly researched, including temperature, pH, and growth medium composition. In this genus, however, no gene involved in GLA formation has been elucidated. Therefore, to identify the function of delta-6 desaturase, Wan et al. cloned it from *C. echinulata* and characterized it with respect to its capacity to direct the synthesis of GLA from LA when heterologously produced in *Pichia pastoris* [85].

Occasionally, *C. echinulata* has been reported as a causative agent of mucormycosis if its spores are inhaled [86]. The fungus has a p450 cytochrome system that is comparable to that of humans, making it a potentially helpful model for studying drug metabolism in the liver [84]. *C. echinulata* has also been employed to transform cortexolone to hydrocortisone [87]. and has been examined for its ability to hydroxylate biphenyl oxide [88]. It can bioabsorb metals, with peak levels of bioabsorption occurring 5 to 15 min after contact with metal [79]. Pekala et al. used different *Cunninghamella* strains to assess the biotransformation pathways of 1-[3-(4-tert-butylphenoxy)- propyl] piperidine (DL76) and novel histamine H3 receptor antagonistin in an in vitro model, and assumed that the oxidation of the methyl group in the tert-butyl moiety leading to the production of a metabolite with alcohol I° characteristics was the most likely metabolic conversion for DL76 [89]. Table 1 summarizes the information regarding genetic manipulation in model Mucoralean fungi discussed above.

## 3. Genetic Tools and Approaches for Genetic Modification in Mucorales

The availability of suitable and efficient genetic modification tools, including reliable and efficient methodology, is not only a fundamental requirement for all molecular and cell biological studies but also for strain improvement strategies using metabolic and genetic engineering. Although many researchers have reported genetic transformation of various Mucorales through targeted gene disruption, gene deletion and the integration of exogenous DNA into the genome of the host organism have remained bottleneck operations in the field [40,99]. Currently, gene silencing with an RNAi-based method is reportedly the most common tool for genetic modification, rather than the typical manipulation of targeted genes by disruption [100,101]. Therefore, this review aims to provide updated information regarding the different genetic manipulation tools and methods in Mucorales and the recent progress made in the field of genetic modification.

### 3.1. Restriction Enzymes

Restriction enzymes are well known as reagents widely used by molecular biologists for genetic manipulation and analysis [102]. They are among the first generation of genome editing tools employed in medical mycology. These enzymes are also frequently used to verify the identity of a genome in order to enable suitable design for modifying it by cutting DNA molecules at specific locations and introducing new genes into the gaps [103]. The discovery of restriction endonucleases has engendered genetic engineering and advancements in molecular techniques in recent research. Restriction enzymes’ mechanism makes them a valuable tool for gene cloning. They have frequently been utilized to genetically manipulate medically important fungi since their discovery in the early 1970s. Restriction enzyme-mediated integration (REMI) has been used to develop mutants in medically important fungi such as *Aspergillus fumigatus*, *Candida albicans*, and *A. nidulans* [104,105]. Restriction enzymes’ ability to generate a sequence of genetic patterns has been used to investigate azole resistance mechanisms in pathogenic fungi such as Aspergillus and *Candida* spp. [106,107]. Moreover, restriction enzymes are used to digest fungal cell walls for protoplast generation [92,108]*,* as the fungal cell wall is made up of glucan, chitin, and mannan. Cell wall components differ among fungal species; hence, different enzymes should be employed in combination to generate protoplasts.

### 3.2. Genetic Markers

Mucorales research is still in its early stages compared with research into other pathogenic fungi. Among the genetically amenable Mucorales, *Mucor* and *Rhizopus* spp. have shown greater drug resistance than others [28]. Conventional genetic modification involves drugs as dominant selection markers; as this is ineffective for these species, our ability to genetically manipulate Mucorales is significantly limited [109]. Another method is the utilization of auxotrophic markers for gene replacement in *Rhizopus* and *Mucor. LeuA* and *pyrG* are traditionally used for genetic manipulation in these two Mucorales. The *leuA* gene is an ortholog of the LEU1 gene in *Saccharomyces cerevisiae,* which codes for an isopropylmalate isomerase involved in the leucine biosynthesis process [110]. The *pyrG* gene is an ortholog of the URA3 gene in *S. cerevisiae,* which codes for the orotidine-5′-phosphate (OMP) decarboxylase involved in *de novo* pyrimidine biosynthesis [7,109]. The *pyrF* gene has been utilized as a selection marker in *Rhizopus; pyrF* is an ortholog of the URA5 gene, which codes for an orotate phosphoribosylatransferase that also participates in pyrimidine biosynthesis [111]. Many researchers have used these two selection markers to complement uracil and leucine auxotrophy in *M. circinelloides* [41,47,48]. For instance, Garcia et al. used the *pyrG* gene in *Mucor* as a reusable marker for successive gene deletions. Mutants with deleted *pyrG* could be selected by using 5-fluoroorotic acid (5-FOA), which was decarboxylated by the *pyrG* gene product, generating the toxic chemical 5-fluorouracil [109]. Mutants lacking the wild-type *pyrG* gene could thus survive on 5-FOA and uracil/uridine-containing media. Any mutants from which the *pyrG* tag was removed can be cultivated with this approach. A second gene can then be deleted from the mutants developed with the first transformation after the marker has been removed.

### 3.3. Random Mutagenesis

Random mutagenesis is the process of using mutagens to modify the DNA. A single mutagen or a combination of two or more mutagens may be applied to the target cells. This is a strong tool for disrupting gene function, generating enzymes, and improving metabolic pathway productivity. It is used to develop genes in vitro via an iterative recombinant generation approach. This strategy has been effectively utilized to overcome challenges in protein engineering when combined with powerful, high-throughput screening or selection tools. Chemical mutagenesis was employed in *R. arrhizus* to develop auxotrophic mutants using N-methyl-N-nitro-N-nitrosoguanidine [112] or to boost L(+)-lactic acid production with diethyl sulphate [113]. Radiation with UV light, gamma radiation with ^60^Co [113], and low-energy ion implantation have all been used to induce random mutations [114]. This technique’s disadvantages include the generation of multiple mutations and the time needed for screening and subsequent selection rounds. Effective screening methods are needed to assess mutants for desired features.

For complementation analysis of carotenogenic mutants, vegetative spore suspensions (approximately 106 spores/mL) of several strains of *M. circinelloides* were treated with N-methyl-N8-nitro-N-nitrosoguanidine. Mutants of the filamentous fungus with altered β-carotene production (genotype car) were identified by inspecting the color of the colonies obtained from mutagenized spores [115]. Similarly, in another study, spores of an *M. circinelloides* auxotrophic strain were subjected to UV radiation (50 mJ/cm^2^) or NTG in conditions that resulted in a 1–5% survival rate in an attempt to understand the underlying regulatory mechanism of carotenogenesis in this fungus via mutagenesis. Mutagenesis and screening resulted in 26 mutants with altered color [23]. Jin et al. used a UV mutated strain of *M. alpina* ME1 and an optimized mycelia aging technique to obtain 19 g/L of ARA production in a 5L fermenter [95]. The overexpression of the ω-3 fatty acid desaturase gene from *Phytophthora parasitica* (PpFADS17) in *M. alpina* suggests its potential as an EPA-producing strain under normal temperatures [96]. Takeno et al. used chemical mutagens such as N-methyl-N′-nitro-N-nitrosoguanidine to screen a uracil-deficient strain of *M. alpina* and found that it had no orotate phosphate ribosyl transferase activity. They then extracted the ura5 gene and used it as the selective gene to create the pDura5 vector [69].

### 3.4. Gene Knockout

**Figure 1:**
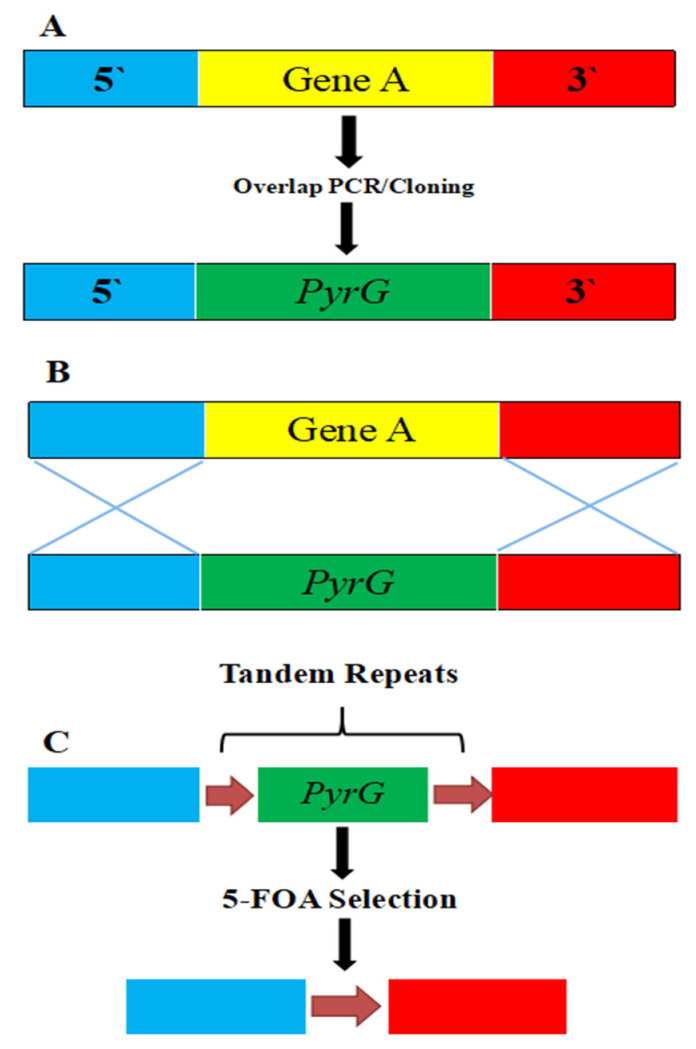
Representation of gene deletion in *Mucor* and *Rhizopus* spp. (**A**) ~1 kb of the 5′ and 3′ end of the target gene is incorporated on their respective sides to the *pyrG* marker gene. This is accomplished via overlapping PCR or cloning methods. (**B**) The deletion construct is delivered into the fungal cells via protoplastic transformation, electroporation, or biolistic transformation for homologous gene replacement. (**C**). To construct a recyclable marker, the *pyrG* gene is flanked on either side with a 237-bp repeat, resulting in the *pyrG-dpl237* marker. The tandem repeats of 237 bp around the *pyrG* gene facilitate the excision of the *pyrG* marker after target gene deletion [28].

In Mucorales, particularly *Mucor* and *Rhizopus* spp., traditional gene deletion constructs have also been designed to knock out a particular gene (Figure 1). To do so, 1-kb DNA fragments located upstream and downstream of a target gene are placed at the 5′or 3′ end of the marker gene (*pyrG* or *leuA*). The deletion construct is created using overlapping PCR or cloning techniques, and then transferred into the fungus via protoplast-mediated transformation, electroporation, or biolistic transformation for gene replacement based on homologous recombination [92,109,116]. The homologous recombination between the gene of interest and the marker is subsequently enhanced by juxtaposing 1-kb segments of the target gene [90]. This traditional gene deletion strategy has contributed to our knowledge of Mucorales. For example, the mechanism of light-sensing in *Mucor* was explained by deleting the related white color gene complex [115]. Similarly, disrupting the *crgA* gene increased the understanding of its negative role in carotenogenesis in *M. circinelloides*. This *crgA* gene is reportedly involved in light-induced carotenoid biosynthesis. This regulatory role was confirmed by the development of a *crgA* null mutant through gene replacement strategies [23,117].

In *M. circinelloides*, the two most advantageous loci *carRP* and *carB* [118,119] are well known for the targeted integration of a gene that codes for three enzyme activities involved in the production of β-carotene. The region 1 kb up- and downstream of *carRP* helped the disruption of this particular gene and the integration of a targeted gene that resulted in white-colored mutant strains which could be easily distinguished from the yellow phenotype of wild-type *M.circinelloides* [37,46,92]. These gene knockout methods have also been used to demonstrate functions of the *sexM* gene in sexual development; of Argnaute genes, RNA-dependent RNA polymerase (RdRP) genes, and Dicer genes in gene silencing; and of calcineurin genes in dimorphism and virulence [8,120]. Similarly, the roles of protein kinase A and factors involved in ADP ribosylation in morphogenesis were described through relevant gene deletions [27,121]. Genes can be knocked out through double crossing-over events, which can help in investigating the loss of a specific gene function or its effect on metabolic pathways. A similar technique was employed in *Rhizopus* to produce a deletion construct, which was then transformed to generate a deletion allele [122]. Ibrahim et al. employed this conventional gene knockout approach to elucidate the role of the high-affinity iron permease gene (FRT1) in iron uptake and pathogenicity [21].

### 3.5. Gene Silencing Using RNA Interference

**Figure 2:**
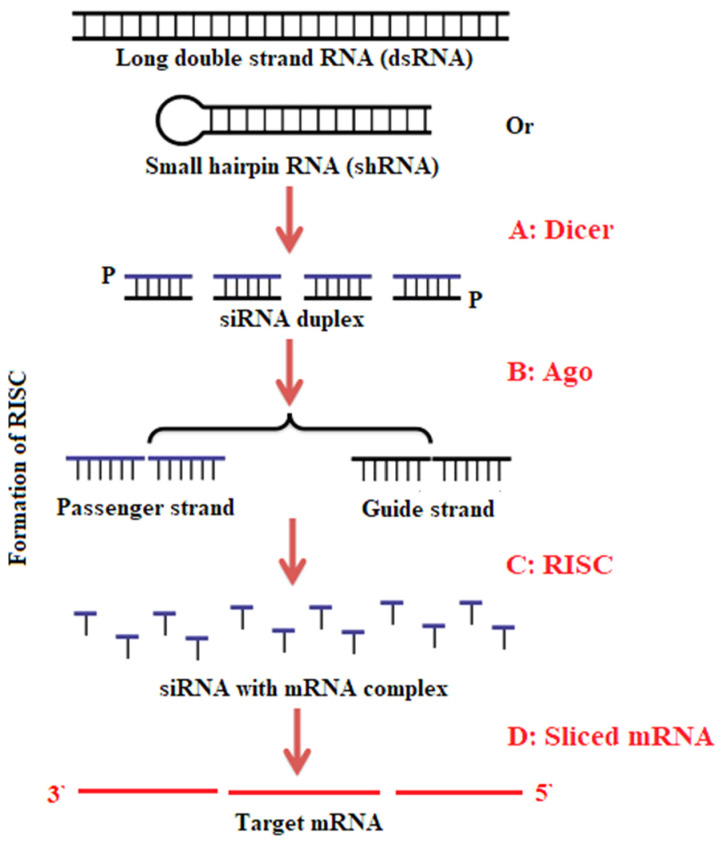
Hypothetical model of the RNAi pathway in fungi. The Dicer ribonuclease III enzyme (DCR) cleaves exogenous dsRNAs into ~21–24 nucleotide siRNAs. The guide siRNA is then loaded onto the major catalytic component, called Argonaute (Ago), and other proteins generating the RISC. The siRNA, along with RISC, complementarily pair with messenger mRNA, resulting in degradation of mRNAs [132].

RNAi is another elegant way to downregulate gene expression. It is a recently developed method in which double-stranded RNA initiates the breakdown of homologous sequences of messenger RNA, resulting in the reduction or complete cessation of translation of related proteins [123]. Several proteins are required for the RNAi machinery: Dicer, Argonaute proteins, and RdRP. The Dicer cuts double-stranded RNA (dsRNA) into double-stranded short interference RNA (siRNA). Argonaute then binds to the siRNA fragments, retaining the single-stranded RNA. The process of silencing involves two different RNA polymerase enzymes in fungi. RdRP1 is the first polymerase that starts the silencing by generating antisense RNA transcripts from the target gene, whereas RdRP2 is the second polymerase that produces new dsRNA molecules from the target mRNA template by strengthening the silencing signal [124]. The RNase III Dicer processes dsRNA, molecules obtained from exogenous sequences to form siRNAs, which are attached to an Argonaute protein inside the RNAi silencing complex (RISC) (Figure 2). These siRNAs are then used as a guide to detect complementary target RNA molecules for gene silencing or degradation [125].

*Mucor* species possess a conserved RNAi pathway that is involved in the post-transcriptional regulation of genes [126,127,128]. Endogenous short RNAs (esRNAs) are produced by the cleavage of double-stranded RNA (dsRNA) and regulate endogenous gene expression in *Mucor* by degrading their associated mRNAs [129]. The identification of two different types of small antisense RNAs associated with silencing was achieved by transforming self-replicating plasmids or vectors involved in the expression of inverted repeat transgenes [126,130]. Short antisense RNAs (21 nt) are only detected in the RNA of spores, whereas long antisense RNAs (25 nt) are only synthesized or detected in the early vegetative growth stage (late-stage).

In one study, gene silencing was triggered with hairpin RNA, which showed that a single Dicer gene (*dcl-2*) was required for gene silencing and vegetative growth [131]. Synthetic siRNAs have also been used to initiate the RNAi response. The product of dcl-2 split the dsRNA molecules into the two types of siRNAs, which were then added to a single Argonaute gene (*ago-1*) to mediate endogenous and exogenous gene silencing in *M. circinelloides* [128].

One investigation of mRNA profiling revealed the role of RNAi machinery in controlling the response to certain environmental signals [42]. In a recent study, RNAi processes reportedly caused epimutations in fungal pathogens by temporarily silencing certain genes in response to stressful stimuli. These epimutations were produced by two interacting RNAi pathways in *M. circinelloides*: the canonical RNAi pathway and a novel RNAi degradative pathway. The second is regarded as a non-canonical RNAi pathway (NCRIP) because it used RdRPs and a novel ribonuclease III-like (R3B2) protein to degrade the targeted transcripts [133]. *M. circinelloides* has been found to have a sort of genetic mutation that contributes to rapamycin and FK506 resistance via the RNAi pathway [101].

**Figure 3:**
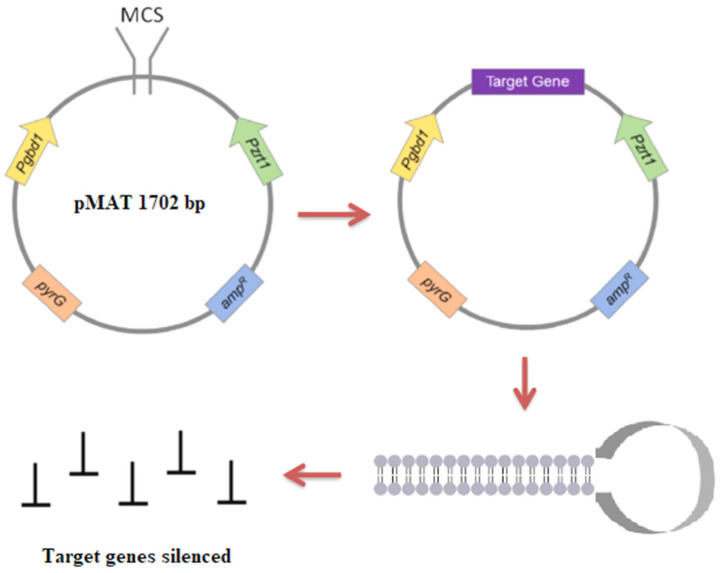
Gene silencing in *Mucor* and *Rhizopus* spp. Two convergent promoters flank a multiple cloning site that can be digested, where a target gene can be placed. *Amp* and *pyrG* act as selectable markers, and the presence of the *carB* gene adjacent to the target gene allows for the selection of white colonies post-transformation. The dsRNA resulting from this self-replicative vector can silence the target gene. Data taken from [28].

The gene silencing mechanism has been extensively investigated and is also used as a molecular tool for genetic modification in this fungus. For instance, Trieu et al. developed a whole-genome silencing library using the silencing vector pMAT1700 [100]. As plasmids can be modified to silence any target gene, this self-replicating plasmid is a useful tool to screen for functional genomics. It usually contains a *pyrG* selection marker and a silencing cassette. The silencing cassette has two convergent promoters (*Pgpdh1* and *Pzrt1*) flanked by a multiple cloning site (MCS) that generates dsRNA that can activate the gene silencing mechanism of *Mucor* and *Rhizopus* spp. (Figure 3). The *carB* gene, which is responsible for carotene synthesis and also functions as a silencing reporter, rests between the two promoters. A vector within the target gene can then be transformed via electroporation. As the *carB* gene is silenced at the same time as the target gene, successful silencing of the cloned genomic segment results in easily visible white colonies. Using this method, researchers discovered two new virulence factors: phospholipase D and myosin 5 [100]. Thus, gene silencing can be used as a tool to screen and identify unique phenotypes or any other characteristics distinct from the wild-type strain, and then functional validation can be conducted using knockdown strains.

A gene silencing system was also employed to genetically manipulate *Rhizopus* as a molecular tool. The genomic sequencing of *R. arrhizus* strain 99–880 suggested the presence of two Argonaute copies, one Dicer, and five *RdRP*-encoding genes. Ibrahim et al. used RNAi to silence the high-affinity iron permease-encoding gene (ftr1) and evaluate its effectiveness. A plasmid construct was produced with sense and antisense (both 450 bp) versions of the *ftr1* gene separated by a spacer element [21]. After the construct was transcribed, a hairpin structure (hpRNA) was generated, starting the RNAi process. This technique successfully silenced the gene, and the iron uptake of the transformants developed through this construct was lowered by approximately 50% [21]. In a similar study, siRNA was used to knockout lactate dehydrogenase (*ldhA* and *ldhB*) genes, which resulted in an 85.7% reduction in the production of lactic acid [98]. To do so, synthetic siRNAs were created for a specific region in the LDHA-encoding gene with the highest sequence similarity to *ldhB.* These 25-nucleotide siRNAs were utilized to transform *R. arrhizus* CCUG 28958 protoplasts. A glucose-related protein (GRP78) was recently revealed to act as an endothelial cell receptor to which Mucorales species might bind during host cell invasion [22]. A CotH3, CotH2 *Rhizopus* mutant was created as a result of this research (RNAi: CotH2,3). The above findings showed that RNAi can be employed to downregulate gene expression. Gene silencing has several advantages over gene knockout in Mucorales, the most important of which is the ability to examine essential genes. It can also compensate for the lack of selection markers in Mucorales.

Takeno et al. increased the FA content of *M. alpina* with RNAi. They were able to silence the 12-desaturase gene, which is important in FA synthesis, and increase the concentration of FAs produced. More crucially, the FA composition was not affected by the RNAi-based silencing [97]. However, the major hurdle for successful heterologous gene expression is genomic integration of the vector DNA. Furthermore, there are no dominant selection markers and few auxotrophic markers present. The effect of siRNA-mediated gene silencing is temporary, making it unsuitable for industrial applications, and it can have unpredictable off-target effects [134].

### 3.6. CRISPR-Cas9 Based Genome Modification

**Figure 4:**
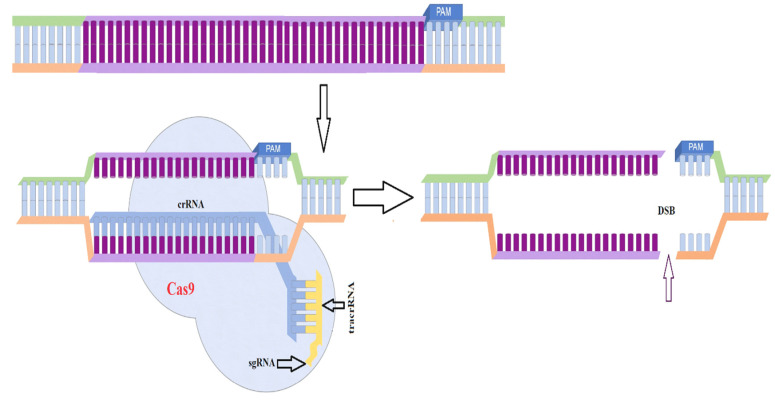
Cas9 induced double-strand break in a target gene. The sgRNA comprises tracrRNA and crRNA. The crRNA is specific to the target DNA, and the Cas9 enzyme creates a double strand break upstream of the PAM motif [28].

Clustered regularly interspaced short palindrome repeats (CRISPR) systems are DNA sequences present in bacteria that protect bacteriophages [135]. The *Streptococcus pyogenes* Type II CRISPR-Cas system has been widely investigated, and CRISPR-associated protein 9 (Cas9) is required for all stages of *S. pyogenes* immunity [136]. The CRISPR-Cas9 genome editing system is a novel and versatile method that has already been used to target and disrupt genes in both yeast and filamentous fungi and to perform gene editing in plants [137], embryos [100], and microorganisms [138,139,140]. The CRISPR-Cas9 system consists of two key components: a single guide RNA (sgRNA) and a Cas9 nickase [141]. The sgRNA is obtained by linking CRISPR RNA (crRNA), which is complementary to a particular part of the target gene, with trans-activating crRNA (tracrRNA). The sgRNA instructs the Cas9 nuclease to create a double-stranded nick with a protospacer adjacent motif (PAM) in the 20-bp target DNA (Figure 4). Chemical changes at specific sites of the sgRNA have also been found to boost the specificity of the Cas9 enzyme(s) and reduce off-target effects [142]. In filamentous fungi, low gene editing frequencies offer a major problem for genetic modification, particularly in *Aspergillus* spp. [139,143], *Neurospora crassa* [144], and *Tichoderma reseei* [145]. CRISPR-Cas9 has been used to solve this problem, and recently gained popularity in the field of genetic manipulation of microorganisms that cause mucormycosis.

CRISPR-Cas9-based genome editing in filamentous fungi can be conducted via a variety of approaches. Multiple plasmids can be constructed and transformed by an in vivo expression vector that contains the Cas9 gene and sequences the encoding parts of the guide RNA (gRNA), including crRNA, tracrRNA, and the protospacer fragment, ensuring their expression. Although these techniques are effective, using expression vectors for CRISPR-Cas9 genome editing has certain disadvantages. In addition, creating the required plasmid expressing cas9 and the proper gRNA can be time-consuming and difficult, and these systems allow the plasmids to persist and replicate after the genome editing event, which can limit the applicability of this approach and generate mutants. These mutants may contain bacterial antibiotic resistance genes and other bacterial sequences utilized for plasmid selection during development that are contraindicated in many uses. Furthermore, the presence of plasmid and foreign DNA over the long term may create off-target effects as well as undesired reorganization and degradation of the exogenous DNA and the genome, which are frequently reported in Mucorales fungi [40,99]. In the plasmid-free method, a ribonucleoprotein (RNP) complex generated by the Cas9 enzyme and in vitro transcribed gRNA is transformed into a cell to engineer the gene of interest. One of the advantages of this approach is that off-target effects can be avoided using this strategy; however, the RNP can be readily degraded. Recently, a novel CRISPR-Cas9 system was designed by Nagy et al. and proved as a tool for site-specific mutagenesis of *M. circinelloides* without the use of plasmids or in vitro RNP synthesis [146]. Their team employed this plasmid-free approach for targeted gene disruption of *carB* and *hmgR2* genes in *M. circinelloides,* resulting in the generation of stable mutants. They directly introduced the guide RNA, Cas9 enzyme, and in the case of homology-directed repair (HDR), the template DNA into the recipient strain [146]. This plasmid-free technique has several advantages over the in vivo strategy, including the elimination of additional cloning processes, the avoidance of off-target effects due to the temporary exposure to Cas9 in transformed cells, and the ability of Cas9 to be active immediately after being transformed into the cells.

Similarly, for targeted gene disruption in *Rhizopus*, a plasmid-based approach was employed by Baldin et al., combining all of the necessary components into a single vector [147]. Based on the well-established fact that polyketide synthase (PKS) is an important enzyme that produces toxins in filamentous fungi [148], and its disruption results in transgenic fungi with a considerable reduction in their harmful effect on their hosts [149], Fuller and coworkers used a CRISPR system for targeting loss of function in PKS to study gene-related functions. They observed high editing efficiency (25–53%) and established that Cas9 expression is useful for *Aspergillus fumigatus* growth and other characteristics [139]. According to several studies, genetic modification using CRISPR-Cas9 in the fungus *A. fumigatus* has been successfully undertaken to demonstrate the importance of various genes in *Aspergillus* [150,151]. For example, Umeyama and coworkers applied CRISPR to replace the cyp51A gene in azole-resistant clinical isolates of *A. fumigatus* [150]. In this investigation, a Cas9-gRNA ribonucleoprotein complex and a donor template were supplied into cells simultaneously, followed by azole susceptibility testing in transformants that revealed enhanced susceptibility due to the replacement of *Ser138* with glycine [150]. More recent studies have also indicated the applications of CRISPR-Cas9 technology for the synthesis of recombinant proteins in *Aspergilli* species [152,153]. These investigations showed that CRISPR-Cas9 performs well in *Aspergillus* research and that components of the system have a wide range of usefulness.

CRISPR-based techniques have been proven beneficial among the various technologies due to several reasons: they allow a simpler design procedure and more economical and faster execution compared with nuclease platforms, making them highly desirable engineering tools for industrially important microorganisms that do not have genetic tools for modification. The CRISPR system’s multiplexing features [154,155,156,157] can help it to perform better in the future in a variety of ways; for example, in the establishment of fungal cell factories for the synthesis of high-value added products and metabolites [157] or novel antifungal bioactive compounds [158], the system can be made more economical by techniques such as genome reduction to reduce undesired products [159] and developing sets of deletion mutants over fungal gene interaction systems, respectively [160].

## 4. Conclusions

Fungi of the phylum Mucoromycota have been reported with various functions and applications in research and industrial and medical fields. Ecologically, Mucoralean fungi represent a diverse group, but ecological roles and geographic distribution remain unidentified for the majority of taxa. Various recent advances have been made in understanding the genetics of Mucoromycota fungi, for instance, construction of recyclable markers for genome manipulation and silencing vectors, gene silencing with RNAi, and the revolution of the CRISPR-Cas9 gene editing system. This review discusses several important genetic modification tools and approaches which are used to manipulate the genome of model organisms of the order Mucorales for useful metabolite production and gene regulatory studies. These genetic tools have been successfully used to obtain valuable bioactive compounds from these industrially important fungi. Though progress has been made by overcoming challenges to genetic manipulation in these species, we are still far from fully understanding the Mucorales order. Genetic manipulation of Mucorales has some limitations due to the limited availability of genetic tools. There is a need to further explore the potential of these fungi and tune beneficial pathways for increased titer and yield of a variety of valuable compounds.

## Figures and Tables

**Table 1:** Representative microorganisms of some selected Mucoromycota and employed engineering strategies for their production of valuable bioactive compounds.

Organism	Gene Name	Strategy Employed	Product Obtained	References
*M. circinelloides* CBS 277.49	Delta 12 and delta 6 desaturase	Overexpression	GLA	[90]
*M. circinelloides*	Delta-6 elongase	Overexpression	DGLA	[46]
*M. circinelloides*	Citrate transporter	Overexpression	44% lipid content elevation	[91]
*M. circinelloides*	Snf-β	Overexpression and knockout	32% lipid content elevation	[92]
*M. circinelloides*	*crgA*	Mutagenesis and deletion of *crgA* gene	4 mg/g β-carotene 268–527 µg/g β-carotene	[23,93]
*M. circinelloides*	*crtS* and *crtR*	Overexpression	190 µg/g canthaxanthin	[48]
*M. circinelloides*	*crtW* and *crtZ*	Co-expression	145–443 µg/g canthaxanthin, 35 µg/g astaxanthin	[47]
*M. circinelloides*	*bkt*	Overexpression	576 µg/g canthaxanthin	[94]
*M. alpina*	gamma linolenic acid elongase	Overexpression	Twofold greater production of ARA	[69]
*M. alpina*	GK and ME1	Co-overexpression	Fatty acid accumulation by 81%	[70]
*M. alpina*	N/A	UV mutagenesis (optimized mycelia ageing technique)	19 g/L of ARA production	[95]
*M. alpina*	ω-3 fatty acid desaturase	Heterologous expression	EPA	[96]
*M. alpina*	G6PD2	Overexpression	1.7-fold rise in total FA production	[60]
	malE2		1.5-fold increase in ARA content	
*M. alpina*	VHb	Heterologous overexpression	eight times more total lipid and ARA	[71]
*M. alpina*	12-desaturase gene	RNA interference	19.02 g/L of ARA	[97]
*M. alpina*	*MaLeuB*	Homologous overexpression	20.2% higher total FA	[54]
*R.* *arrhizus*	*ldhA* and *ldhB*	Small interfering RNA	15.4% increment in ethanol yield	[98]
*C. echinulata*	*-*	Grown on tomato waste and potato starch	1018 and 540 mg/L of GLA, respectively	[81,82]
